# Artificial intelligence analysis of OCT biomarkers to predict visual outcomes following vitrectomy for epiretinal membrane

**DOI:** 10.1186/s40942-025-00735-9

**Published:** 2025-10-14

**Authors:** Lorenzo Ferro Desideri, Leandro Hinrichsen, Nina Eldridge, Hung-Da Chou, Yu-Chieh Chang, Yousif Subhi, Raphael Sznitman, Martin Zinkernagel, Rodrigo Anguita

**Affiliations:** 1 Department of Ophthalmology, Inselspital, Bern University Hospital, University of Bern, Freiburgstrasse 15, Bern, CH-3010 Switzerland; 2 Bern Photographic Reading Center, Inselspital, Bern University Hospital, University of Bern, Bern, Switzerland; 3 Faculty of Medicine, University of Bern, Murtenstrasse 11, Bern, CH-3008 Switzerland; 4 ARTORG Research Center Biomedical Engineering Research, University of Bern, Bern, Switzerland; 5 Department of Ophthalmology, Chang Gung Memorial Hospital, Linkou Medical Center, Taipei, Taiwan; 6 Department of Ophthalmology, Rigshospitalet, Glostrup, Denmark; 7 Department of Clinical Research, University of Southern Denmark, Odense, Denmark; 8 Department of Clinical Medicine, University of Copenhagen, Copenhagen, Denmark; 9 Moorfields Eye Hospital NHS Foundation Trust, London, UK

**Keywords:** Epiretinal membrane, Artificial intelligence, OCT biomarkers, Visual prediction

## Abstract

**Background:**

Preoperative optical coherence tomography (OCT) biomarkers may help predict visual outcomes after idiopathic epiretinal membrane (ERM) surgery. Artificial intelligence (AI) enables automated, quantitative analysis of retinal structure, potentially improving prognostication.

**Methods:**

In this multicenter, retrospective study, patients with idiopathic ERM who underwent pars plana vitrectomy (PPV) were included. Preoperative OCT volume scans were analyzed using an AI-based platform (Discovery OCT Biomarker Detector; RetinAI AG) to quantify retinal layer thicknesses and fluid biomarkers within the central 1 mm Early Treatment Diabetic Retinopathy Study (ETDRS) grid. Extracted features included outer nuclear layer (ONL), combined photoreceptor and retinal pigment epithelium complex (PR + RPE), retinal nerve fiber layer (RNFL) thickness, and intraretinal fluid (IRF) volume. A random forest classifier was used to evaluate the importance of these biomarkers in predicting 12-month best-corrected visual acuity (BCVA), categorizing patients as significant improvers (≥ 0.2 logMAR gain) or minimal/non-responders.

**Results:**

A total of 71 eyes were analyzed. Mean BCVA improved from 0.51 ± 0.41 to 0.25 ± 0.33 logMAR at 12 months postoperatively (*P* < 0.001). Thinner preoperative ONL thickness was strongly associated with worse final BCVA (*r* = − 0.54), while thicker RNFL (*r* = 0.28) and greater IRF volume (*r* = − 0.26) were also linked to poorer outcomes. The random forest model achieved an area under the curve (AUC) of 0.71 for predicting visual improvement, identifying PR + RPE thickness, RNFL thickness, and ONL thickness as the most influential predictors.

**Conclusions:**

Preoperative AI-derived OCT biomarkers, particularly indicators of outer retinal thinning and inner retinal thickening, are associated with limited visual recovery following ERM surgery. Integration of automated biomarker analysis into preoperative assessment may help identify patients at higher risk of suboptimal postoperative vision, informing surgical decision-making and patient counseling.

## Background

Idiopathic epiretinal membrane (ERM) is a retinal disorder characterized by the formation of a thin fibrocellular layer over the macula, often associated with anomalous vitreoretinal separation, leading to retinal distortion and subsequent visual impairment [1, 2] with prevalence estimates ranging from 2% to 34% in individuals aged 50 years and older [3]. ERM is typically categorized into early forms (cellophane macular reflex) and advanced forms (preretinal macular fibrosis), reflecting increasing degrees of fibrocellular proliferation and retinal traction [4]. While in most of the patients (66%-90%) an improvement in visual function has been reported to occur even after 3 years from surgery, in a minor percentage of them visual recovery may be unsatisfactory [5]. Several studies have described the presence of different preoperative optical coherence tomography (OCT) features in ERM in association with postoperative visual outcomes, including photoreceptor integrity, the presence of an ectopic inner foveal layer (EIFL), inner nuclear layer thickness, and photoreceptor outer segment length [6, 7]; however, the predictive value of these OCT biomarkers still remains variable, highlighting the need for more robust evidence [8].

Recently, Govetto et al. proposed an OCT-based staging system (stages 1–4) that reflects progressive disruption of foveal architecture and has been shown to predict both monocular and binocular functional outcomes [9]. This classification underscores the clinical relevance of standardized structural grading, which may gain additional value when combined with artificial intelligence (AI) driven OCT biomarker extraction platforms.

In recent years, artificial intelligence (AI) has been increasingly used in order to improve diagnostic capability, prognostication, and aiding treatment algorithms in the retinal diseases [10, 11]. AI-developed algorithms can extract and quantify retinal biomarkers with higher reproducibility than manual evaluation, offering potential for improved prognostic assessment in vitreoretinal diseases, including ERM [12]; however, the role of AI in predicting visual outcomes after surgery in vitreoretinal field still remains largely unexplored.

This study aimed to use AI-driven OCT analysis to identify preoperative biomarkers in patients undergoing pars plana vitrectomy (PPV) for ERM. Furthermore, we performed predictive models with machine learning algorithms to correlate preoperative OCT biomarkers in ERM with postoperative visual acuity.

## Methods

### Study design and participants

This multicenter, retrospective study included patients diagnosed with idiopathic ERM who underwent PPV. Clinical and imaging data were collected from two centers: (1) Department of Ophthalmology, Inselspital, Bern University Hospital, (Switzerland) and (2) Department of Ophthalmology, Chang Gung Memorial Hospital, Linkou Medical Center (Taiwan).

This study was conducted in accordance with the Declaration of Helsinki, and institutional review board approval was obtained. Informed consent was acquired from all participants prior to data inclusion.

Patients were included if they had both preoperative and postoperative functional and morphological data, including OCT scans, with a minimum follow-up of 12 months. Clinical and imaging data were collected preoperatively (within 2 months before surgery) and at least 12 months after surgery.

Exclusion criteria included a history of retinal detachment, uveitis, diabetic retinopathy (DR), age-related macular degeneration, or other retinal diseases that could affect visual outcomes. Patients with significant media opacities at baseline or during the follow-up, including advanced cataract potentially affecting OCT image quality or visual acuity assessment, were also excluded from the analysis.

### Visual function

Best-corrected visual acuity (BCVA) was assessed preoperatively and postoperatively using Snellen charts and converted to logarithm of the minimum angle of resolution (logMAR) for statistical analysis. As described in our previous study, patients were arbitrary categorized into two groups based on their visual improvement at 12 months: (1) Improvement Group (BCVA improvement ≥ 0.2 logMAR) and (2) No Improvement Group (< 0.2 LogMAR change) [12].

### OCT imaging and AI-based analysis

**Fig. 1:**
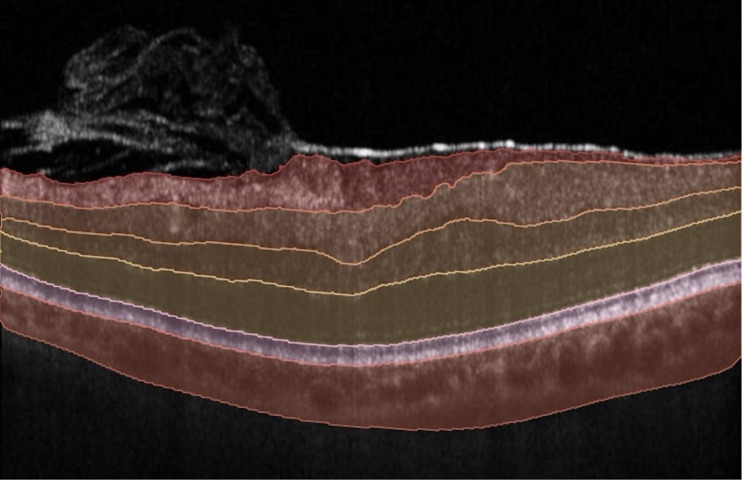
Preoperative OCT scan of a patient with an epiretinal membrane (ERM). The automated segmentation highlights retinal and choroidal layers, clearly demonstrating retinal distortion typical of ERM

Spectral-domain OCT (SD-OCT) images were acquired using the Spectralis SD-OCT system (Heidelberg Engineering, Heidelberg, Germany). The imaging protocol included a 49 B-scan volume centered on the fovea with a resolution of 496 × 512 pixels per B-scan. Preoperative and postoperative OCT volumes were analyzed using an AI-developed software (Discovery OCT Biomarker Detector; RetinAI AG, Bern, Switzerland), which performed automatic segmentation of retinal layers and quantitative biomarker analysis. As reported in our previous work [12], the software provided measurements of retinal layer thicknesses, including the outer nuclear layer (ONL), photoreceptor and retinal pigment epithelium (PR + RPE), ganglion cell layer (GCL), and overall retinal thickness (RT). Additionally, the software identified and quantified the presence and probability of hyperreflective foci (HF) and the quantified volumes of intraretinal fluid (IRF), and subretinal fluid (SRF), as we previously described [12]. Only the foveal ETDRS grid section C1 (1 mm x 1 mm) was included in the analysis. This specific section was selected based on its optimal performance during feature selection for machine learning algorithms and to avoid overfitting of the model. In this software the images are uploaded, and the quantitative output of biomarkers is immediately provided for the user. An example of the automatic segmentation is provided in the figure (Fig. 1). Segmentation quality was reviewed independently by two experienced ophthalmologists (LFD and RA). In cases where automated segmentation errors were detected (> 5% of scans), manual re-segmentation was performed to ensure accuracy of the quantitative outputs.

### Statistical analysis

Statistical analysis was performed using Python version 3.12 with SciPy library (version 1.14). Normality of data distribution was assessed using the Shapiro-Wilk test. Differences in retinal layer thicknesses, intraretinal fluids and biomarker presence between preoperative and postoperative scans were analyzed using paired t-tests. Correlations between OCT features and final BCVA were assessed using Pearson correlation coefficients. Correction for multiple hypothesis testing was done using Benjamini-Hochberg and a false discovery rate of 5%. A multivariate analysis was performed using a random forest classifier model using the scikit-learn library (version 1.5) to determine the most predictive factors for final visual outcomes. Biomarker presence and intraretinal fluids were excluded from the feature set to prevent overfitting. Further feature selection was performed using correlation analysis and variance inflation factor (VIF) to mitigate covariance among predictors. The model was trained using default hyperparameter settings, including 100 trees in the ensemble. The dataset was randomly split into training set (80%) and a test set (20%) using stratified sampling to maintain class distribution. Model performance was evaluated using area under the curve (AUC), accuracy, precision, recall, and feature importance scores (FIS). To minimize overfitting, a 10-fold cross-validation process was applied, with accuracy and AUC monitored across training and test folds to confirm stability of results. A p-value < 0.05 was considered statistically significant.

### Study outcomes

The primary outcome was the correlation between 12-month BCVA and AI-extracted preoperative OCT biomarkers within the central 1-mm ETDRS zone. The secondary outcomes included pre- to postoperative changes in OCT-derived parameters and the predictive performance of machine-learning models in classifying visual improvement (≥ 0.2 vs. < 0.2 logMAR gain).

## Results

### Demographic features

Overall, OCT scans from 122 patients operated for ERM were reviewed and, among them, 71 patients were considered eligible with complete clinical and imaging data. Among them, 52% (*n* = 37) were females and 91% (*n* = 69) were left eyes. Average age was 67.4 (± 8.2) and 76.3% of the subjects (*n* = 58) were pseudophakic at baseline and this proportion did not change during the follow-up period, which was on average 12.4 ± 0.7 months. Furthermore, surgical posterior vitreous detachment (PVD) was induced in 39.5% (*n* = 30) of them. Mean preoperative BCVA in the study subjects was 0.51 LogMAR (± 0.41) and improve to 0.25 LogMAR (± 0.33) after 12 months (*p* < 0.001). Complete demographic and clinical data are provided in the table (Table 1).

**Table 1:** Demographic and clinical features of the study population at baseline

Variable	*n* (%)
**Total Patients**	71 (100)
**Sex**	
Female Male	37 (52.1) 34 (47.9)
**Laterality**	
Left Eye Right Eye	69 (91.5) 6 (8.5)
**Mean Age (years**,** ±SD)**	67.4 (± 8.2)
**Diabetes**	7 (9.2)
**High Myopia**	6 (8.5)
**Lens Status**	
Phakic Pseudophakic	18 (23.7) 58 (76.3)
**Surgical Procedure**	
ERM Peeling Alone ERM + ILM Peeling	6 (8.5) 70 (91.5)
**Surgical PVD Induction**	
No Yes	46 (60.5) 30 (39.5)
**Visual Acuity (LogMAR**,** ±SD)**	
Preoperative BCVA Postoperative BCVA (12 M)	0.51 (± 0.41) 0.25 (± 0.33)

BCVA = Best-corrected visual acuity; ERM = Epiretinal membrane; PVD = Posterior vitreous detachment; SD = Standard deviation

### OCT retinal thickness and volumes

Retinal layers analysis revealed a significant reduction in mean RNFL thickness, which decreased from 32.23 ± 20.31 μm preoperatively to 24.14 ± 6.21 μm (*p* = 0.0019) after 12 months.

Similarly, mean GCL + IPL thickness significantly decreased from 84.74 ± 30.04 μm to 73.68 ± 25.02 μm (*p* = 0.0392), while INL + OPL thickness also showed a reduction from 72.92 ± 26.38 μm to 64.29 ± 21.76 μm (*p* = 0.0649) after 12 months. Mean ONL thickness slightly decreased from preoperatively 110.47 ± 13.50 μm to 108.05 ± 18.40 μm (*p* = 0.4693), and PR + RPE thickness declined from 68.82 ± 7.26 μm to 66.60 ± 7.90 μm (*p* = 0.1463) after the 1-year follow-up period. The overall mean RT thickness showed a marked significant decrease from 436.55 ± 81.83 μm to 357.43 ± 84.47 μm (*p* < 0.0001) and by contrast CC + CS thickness slightly increased from 204.79 ± 55.87 μm to 212.57 ± 54.67 μm (*p* = 0.4754).

Retinal volumetric analysis indicated a significant reduction in average IRF after 12 months, which decreased from 25.35 ± 45.89 nL to 5.34 ± 14.04 nL (*p* = 0.0007). Mean SRF volume also decreased from 1.69 ± 4.16 nL to 0.47 ± 2.11 nL (*p* = 0.0400) after 12 months.

### Univariate analysis

**Fig. 2:**
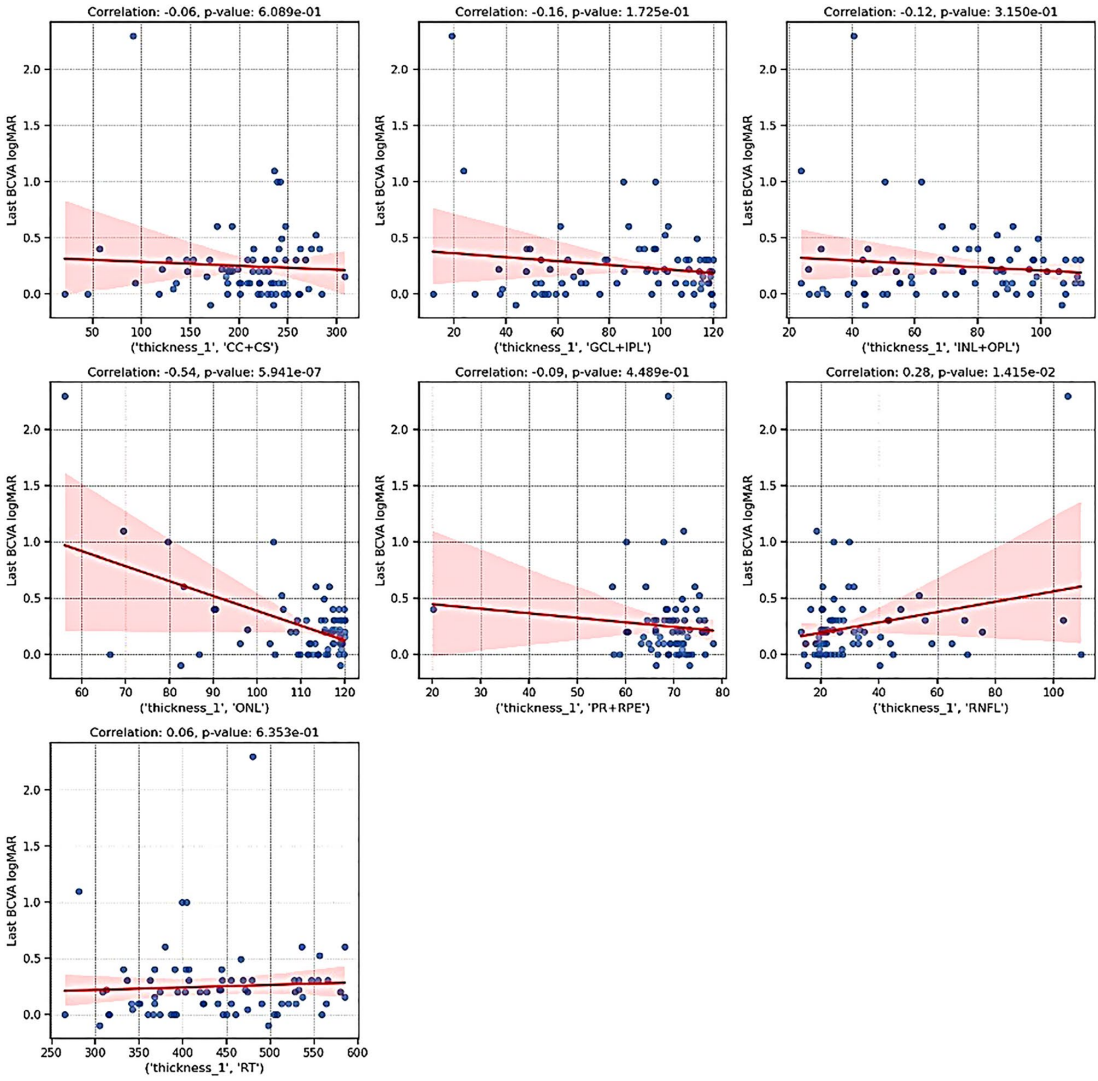
Univariate linear regression between preoperative retinal layers and 12-month visual acuity. A significant negative correlation between outer nuclear layer thickness and a weaker positive correlation for retinal nerve fiber layer and final visual acuity is represented in the scatter plots graphs

Univariate linear regression analysis in relation to 12-month BCVA showed the preoperative ONL thickness was the strongest negative parameter associated with final BCVA (*r* = -0.54, *p* < 0.001), while RNFL thickness demonstrated a weaker positive correlation (*r* = 0.28, *p* = 0.014). Higher preoperative HF presence (*r* = 0.42, *p* < 0.001) was also associated with worse visual outcomes. Other biomarkers, including RT, GCL + IPL, and INL + OPL thicknesses, showed no significant correlation with final BCVA (Fig. 2).

When correlating preoperative OCT features with the change in BCVA univariate logistic regression revealed several significant associations. Preoperative ONL thickness showed a positive correlation with the visual improvement subgroup (*r* = 0.23, *p* = 0.049) after 12 months. Conversely, higher preoperative IRF volume was negatively correlated with improvement (*r* = -0.26, *p* = 0.024). Other biomarkers, such as RNFL thickness (*r* = -0.11, *p* = 0.36) and RT (*r* = -0.13, *p* = 0.26), did not show significant correlations with delta BCVA (Table 2).

**Table 2:** Univariate logistic regression analysis between preoperative OCT retinal layers and biomarkers and the delta of change in BCVA between pre-and postoperative visits expressed in LogMAR

OCT Feature	Pearson correlation	*P*-value
CC + CS Thickness (µm)	0,06676435	0,566623153
GCL + IPL Thickness (µm)	0,004689517	0,967929753
INL + OPL Thickness (µm)	0,044786315	0,700859804
ONL Thickness (µm)	0,226877967	0,048737751
PR + RPE Thickness (µm)	0,075051169	0,519350405
RNFL Thickness (µm)	-0,10687912	0,358124739
RT Thickness (µm)	-0,13029889	0,261911673
IRF (nL)	-0,25962132	0,023524877
SRF (nL)	-0,18742793	0,104957194
HF Probability	-0,17731261	0,125441838

CC + CS = Choriocapillaris and Choroid-Sclera Complex; GCL + IPL = Ganglion Cell Layer plus Inner Plexiform Layer; HF = Hyperreflective Foci; INL + OPL = Inner Nuclear Layer plus Outer Plexiform Layer t; IRF = Intraretinal Fluid; ONL = Outer Nuclear Layer; PR + RPE = Photoreceptor and Retinal Pigment Epithelium complex; RNFL = Retinal Nerve Fiber Layer RT = Retinal Thickness; SRF = Subretinal Fluid

### Machine learning analysis

**Fig. 3:**
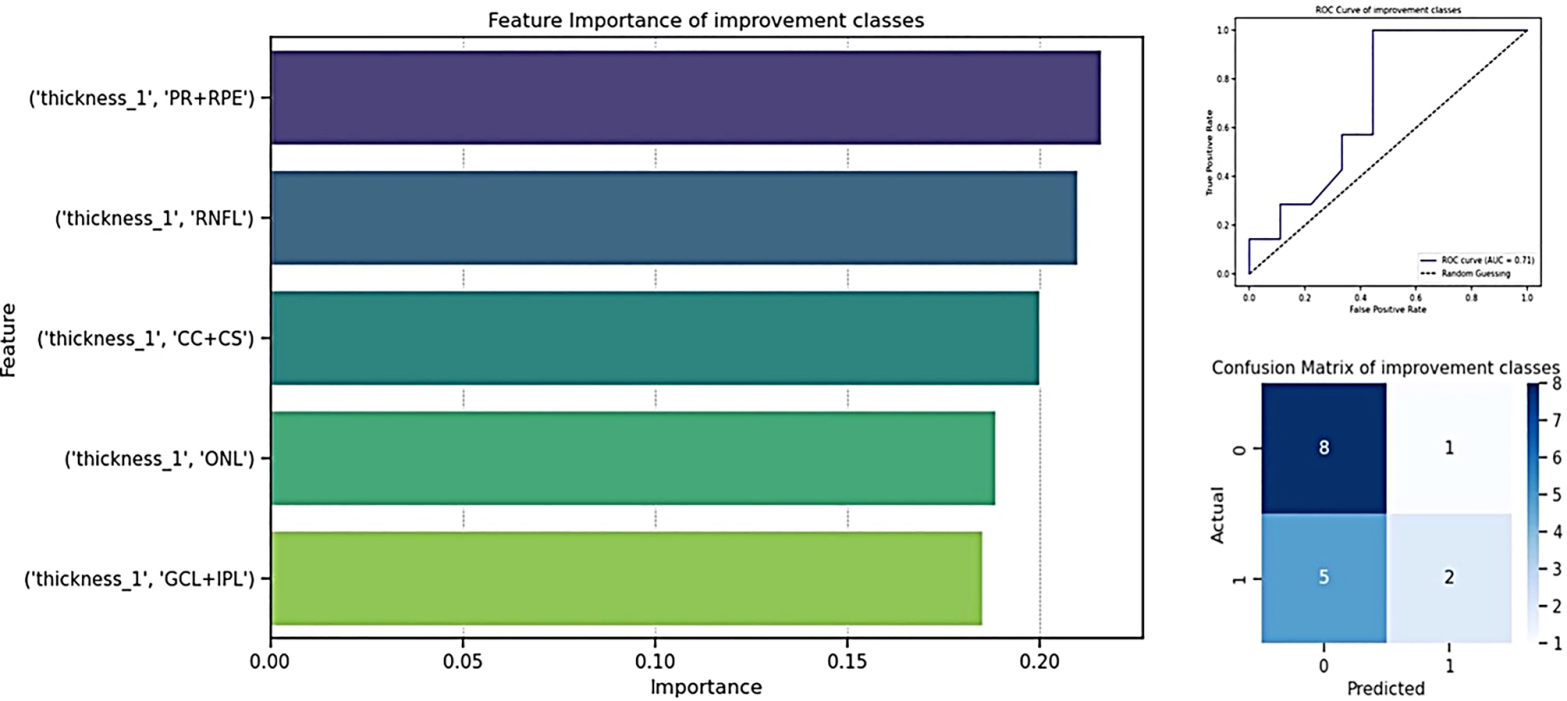
Feature importance and model performance for predicting visual improvement. Prediction of 12-month visual acuity categorized in 1) visual improvement (≥ 0.2 logMAR) and 2) no improvement (< 0.2 logMAR). The figure shows the feature importance of retinal layer thicknesses, with PR+RPE thickness being the most influential, followed by RNFL, CC+CS, ONL, and GCL+IPL. The model’s performance is illustrated through a confusion matrix and receiver operator characteristic curve, highlighting its ability to classify visual improvement outcomes

From the seven retinal layers, including the overall retinal thickness, only 5 were used to train the model. Both the overall retinal thickness and the INL and OPL layer had high correlation values (0.92 and 0.8) and VIF values (76.26 and 69.16) and were therefore excluded. The random forest classifier for distinguishing patients with and without visual improvement from preoperative OCT features had a ROC curve of 0.71. The model scored an accuracy of 0.62 with a precision of 0.64 and a recall of 0.59. FIS analysis revealed that PR + RPE thickness was the most influential predictor (importance = 0.216), followed by RNFL thickness (importance = 0.210), CC + CS thickness (importance = 0.200), ONL thickness (importance = 0.189), and GCL + IPL thickness (importance = 0.185) (Fig. 3).

## Discussion

Prediction of visual acuity is fundamental for counseling patients with ERM prior to surgery in order to establish realistic expectations and assist in appropriate patient counseling. This study used a machine learning analysis to predict visual outcomes from OCT-automated segmentation after 1 year from ERM surgery. We found that a thinner preoperative ONL and PR-PRE complex, were associated with worse BCVA at 12 months; furthermore, the presence of thicker RNFL and GCL + IPL at baseline correlated with poorer functional outcomes.

Several studies in fact have focused on describing preoperative OCT biomarkers in prognosticating postoperative visual function after ERM surgery [13, 14]. Together with the inner retina, including the presence of an ectopic inner foveal layer (EIFL), an inner retinal regularity and GCL-IPL thickness ratio, the outer retina, comprehensive of the integrity of ellipsoid zone (EZ), interdigitation zone (IZ) and the photoreceptor out segment length (PROS) have been shown to display a crucial role in the prediction of BCVA [4, 15, 16].

Most of the previous studies on ERM focused on the role of PR + RPE layer integrity, including PROS length and IZ/EZ status. The EZ is a hyper-reflective band in the outer retina representing the ellipsoid portion of the photoreceptor inner segment, while the IZ, also known as the cone outer segment tips (COST) line, is the hyper-reflective line between the EZ and the RPE, marking the apices of the outer cone segments encased by RPE cell tips [17]. Fernandes et al. demonstrated in multivariate analysis that IZ disruption was a more significant contributor to final BCVA, suggesting that patients operated for ERM with intact EZ may have poorer visual outcomes if the IZ has been damaged. Other studies focused on the PROS length, which is the distance between the RPE and EZ zone, and it has been proven to be more reliable than IZ and EZ integrity assessment especially in presence of artifacts like intraretinal cysts and cataract [18]. Kinoshita et al. revealed after 24 months that a better BCVA correlated with a thicker PROS at baseline [19]. These findings are in line with our study, where we found that a thinner baseline PR + RPE layer (anatomically corresponding to PROS length definition) was associated with worse visual outcomes. Despite certain similarities, two critical differences stand out and warrant further discussion. Firstly, while previous study measured manually the PROS length, an automated measurement tool based on an established AI platform, ensuring objectivity and consistency in evaluation of OCT biomarkers in ERM pathology.

Secondly, the ONL was included as a factor in our BCVA prediction. Given the lack of preclinical models investigating cellular alterations in the outer retinal layers, other in vivo imaging studies provided more hints on the possible role of the ONL for predicting visual outcomes in ERM. For instance, an adaptive optics OCT (AO-OCT) study by Ishikura et al. showed that ERM exerts vertical traction via Müller cells, leading to increased cone nuclei density and distortion of the outer retina, including the ONL [20]. Their AO-OCT imaging and schematic models showed that these forces affected the ONL structure through mechanical compression [20]. A previous study of their group by AO-OCT showed that in ERM also the Muller cells are more vertically distributed in the ONL layers, which is on average thicker in ERM as compared with healthy subjects due to these tractional forces [21]. In contrast, our findings reveal that a thicker ONL correlates with better visual acuity, suggesting that a preserved ONL, rather than traction-induced compaction, is a marker of photoreceptor integrity. This may support the concept that ONL thickness, when not altered by pathological traction, reflects healthier retinal architecture and better functional outcomes, although the relationship between ONL thickness, cell nuclei density, and visual prognosis calls for further investigation.

Contrarily, we described that the inner retina, with RNFL foremost and GLC + IPL thickness showed a moderate positive correlation with worse BCVA at 12 months, meaning that when these layers are thicker at baseline, poorer visual function can be expected after ERM surgery.

This may reflect the extent of ERM-induced centripetal traction, which displaces inner layers toward the fovea and leads to reactive gliosis and Müller cell activation, contributing to both mechanical distortion and tissue hypertrophy [16, 22]. In this regard, the DREAM study demonstrated that severe disorganization of retinal inner layers (DRIL), including RNFL and GCL layers, correlated with reduced postoperative visual gain in ERM patients, confirming that inner retinal integrity is essential for functional recovery. Mild moderate correlation between preoperative foveal and perifoveal RNFL thickness and postoperative visual acuity has been demonstrated, with preoperative INL thickness playing a greater effect on final visual function, speculating a possible ERM-induced microvascular damage at this level leading to neuronal deterioration, including damage to nerve fibers and cellular body of bipolar, amacrine cells and Muller cells [23]. In line with our findings, previous studies reported that thinner baseline GCL/IPL layer predicted better visual recovery after surgery, implying that eyes with less preoperative inner retinal damage retain better potential for functional improvement [24].

In this perspective, our AI-assisted analysis and random forest model further highlighted the importance of the inner retinal health at baseline, not only as a marker of disease severity, but also as a predictor of the ability of the retinal tissue to recover after traction release. This indicates that a more integrated approach may be needed, where inner and outer retinal OCT biomarkers are considered together to fully understand postoperative functional potential.

Lastly, the role of the choroid remains not fully understood in the visual function prognosis. While we found that in univariate analysis there was no significant correlation, multivariate analysis identified CC + CS as the third most prominent feature in the predictive model, highlighting that that the choroid, despite a weak standalone effect, may exert its influence primarily through interactions with other altered retinal layers. In this regard, Michalewska et *al*. revealed that patients with preoperative choroidoscleral irregularity index (CSIi) tended to have a faster postoperative BCVA recovery after ERM surgery [25]. Differently, Doguizi et al. did not report any significant correlation between ERM severity at baseline and choroidal thickness [26]. As from existing evidence is difficult to draw consistent conclusions on the role of choroid in ERM visual prognosis, further studies focusing on this specific layers might be warranted to provide more detailed explanation on the complex interplay between choroidal and retinal layers in this disorder.

Limitations of our study to acknowledge are its retrospective design and the relatively small sample; however, unlike previous studies employing deep learning models that relied primarily on image-level features to predict anatomical or functional outcomes in ERM surgery [27–29], our approach used AI-derived quantitative OCT biomarkers directly incorporated into a machine learning prediction model. This allows for a more interpretable analysis, where individual retinal layer thicknesses and pathological features contribute directly to the prediction model, providing not only prognostic information but also potential pathophysiological insights into retinal microstructural alterations and recovery mechanisms. Such an approach may help overcome the “black box” nature of deep learning, facilitating better integration into clinical workflows [30]. In addition, variations in surgical technique may have occurred and surgeon-related factors were not accounted for in our analysis. Although age, lens status, diabetes, high myopia, and surgical factors (ILM peeling, PVD induction) were balanced and not strongly associated with final BCVA, unmeasured effects cannot be excluded. Incorporating these variables, together with baseline BCVA, into larger multivariable analyses will better account for residual confounding.

In conclusion, this study demonstrates the promising role of AI-driven OCT analysis and machine learning in predicting visual outcomes after vitrectomy for ERM. The findings revealed that thinner preoperative ONL and PR + RPE layers are linked to worse visual results, while thicker retinal nerve fiber layer RNFL correlated with poorer outcomes. These insights highlighted the importance of using an AI-assisted automatized approach to evaluate OCT biomarkers, potentially a more detailed diagnostic and prognostic frameworks in patients undergoing ERM surgery.

## Data Availability

The datasets generated and/or analyzed during the current study are not publicly available due to institutional data protection policies and patient privacy regulations but are available from the corresponding author (LFD) on reasonable request.
